# NeurostimML: a machine learning model for predicting neurostimulation-induced tissue damage

**DOI:** 10.1088/1741-2552/ad593e

**Published:** 2024-06-27

**Authors:** Yi Li, Rebecca A Frederick, Daniel George, Stuart F Cogan, Joseph J Pancrazio, Leonidas Bleris, Ana G Hernandez-Reynoso

**Affiliations:** 1 Department of Bioengineering, The University of Texas at Dallas, Richardson, TX, United States of America; 2 Center for Systems Biology, The University of Texas at Dallas, Richardson, TX, United States of America; 3 Phil and Penny Knight Campus for Accelerating Scientific Impact, University of Oregon, Eugene, OR, United States of America; 4 Department of Computer Science, The University of Texas at Dallas, Richardson, TX, United States of America; 5 Department of Biological Sciences, The University of Texas at Dallas, Richardson, TX, United States of America

**Keywords:** neuromodulation, Shannon equation, machine learning, safe stimulation

## Abstract

*Objective*. The safe delivery of electrical current to neural tissue depends on many factors, yet previous methods for predicting tissue damage rely on only a few stimulation parameters. Here, we report the development of a machine learning approach that could lead to a more reliable method for predicting electrical stimulation-induced tissue damage by incorporating additional stimulation parameters. *Approach*. A literature search was conducted to build an initial database of tissue response information after electrical stimulation, categorized as either damaging or non-damaging. Subsequently, we used ordinal encoding and random forest for feature selection, and investigated four machine learning models for classification: Logistic Regression, K-nearest Neighbor, Random Forest, and Multilayer Perceptron. Finally, we compared the results of these models against the accuracy of the Shannon equation. *Main Results*. We compiled a database with 387 unique stimulation parameter combinations collected from 58 independent studies conducted over a period of 47 years, with 195 (51%) categorized as non-damaging and 190 (49%) categorized as damaging. The features selected for building our model with a Random Forest algorithm were: waveform shape, geometric surface area, pulse width, frequency, pulse amplitude, charge per phase, charge density, current density, duty cycle, daily stimulation duration, daily number of pulses delivered, and daily accumulated charge. The Shannon equation yielded an accuracy of 63.9% using a *k* value of 1.79. In contrast, the Random Forest algorithm was able to robustly predict whether a set of stimulation parameters was classified as damaging or non-damaging with an accuracy of 88.3%. *Significance*. This novel Random Forest model can facilitate more informed decision making in the selection of neuromodulation parameters for both research studies and clinical practice. This study represents the first approach to use machine learning in the prediction of stimulation-induced neural tissue damage, and lays the groundwork for neurostimulation driven by machine learning models.

## 1. Introduction

Neuromodulation of the central and peripheral nervous systems has been widely used to restore function after injury or disease. Generally, neuromodulation by electrical stimulation is used as a last line of treatment for refractory or intractable conditions that have no other alternatives to achieve relief or restore function. Some of the most successful examples of neuromodulation include deep brain stimulation (DBS) to treat Parkinson’s disease and essential tremor [1], cochlear implants to restore hearing [2], vagus nerve stimulation to treat epilepsy [3], reduce inflammation [4], and to improve motor function after stroke or spinal cord injury when paired with rehabilitation [5, 6], sacral nerve stimulation to treat overactive bladder and fecal incontinence [7, 8], and spinal cord stimulation to treat paralysis and chronic pain [9, 10]. Despite the many successful implementations of electrical neuromodulation to treat disease and restore function, the potential damage that electrical stimulation can cause to neural tissues remains a concern. The evaluation of neural stimulation safety relies on only a limited set of parameters derived from a set of important yet limited systematic studies [11–17]. Much of the reason for the inability to precisely define when electrical stimulation-induced tissue damage will occur lies in the complexity of processes occurring during neuromodulation, and the large number of stimulation waveform parameters available to modulate in addition to electrochemical and physiological variables. In addition, electrode selection and design can influence electric field distribution in the tissue, result in non-reversible charge-transfer reactions at the electrode-tissue interface, and electrode dissolution, which may also lead to stimulation-induced tissue damage. Several experimental studies have systematically investigated the relationship between stimulation parameters and the potential for tissue damage [12, 18, 19]. However, the development of predictive models for neural tissue damage has been hindered by the relatively limited availability of experimental data when compared to the number of variables involved.

The most well-known and accepted mathematical relationship for predicting stimulation-induced tissue damage is the Shannon equation [20] that was published in 1992 ‘as an initial framework for designing future animal safety studies’. The model describes a logarithmic two-dimensional mathematical boundary to separate likely damaging from likely non-damaging stimulation parameters based on data from stimulation damage observed in cat cortex using epidural macroelectrodes with geometric surface areas (GSA) ranging from 0.01 to 0.5 cm^2^. The equation relates the charge per phase and the charge density (charge per unit electrode surface, delivered within the leading phase of a charge-balanced stimulus pulse) (equation (1)).

\begin{align*}k = {\log _{10}}\left( {{\text{Charge density}}} \right) + {\log _{10}}\left( {{\text{Charge}}} \right).\end{align*}

For the Shannon equation, a proposed limit between damaging and non-damaging was set at a range between *k* = 1.5 and *k*= 2.0, where *k*-values greater than the proposed limit are considered damaging. Furthermore, an upper limit of 30 *μ*C cm^−2^ was approved using macroelectrodes of 0.06 cm^2^ by the FDA for DBS stimulators. However, there are neuromodulation cases requiring descriptions outside the capabilities of the Shannon model. For example, recent intracochlear stimulation at levels above 100 *μ*C cm^−2^ have shown no reduction in auditory neuron function despite chronic stimulation above the Shannon equation threshold [21]. In another example, microelectrodes with geometric surfaces areas less than 2000 *μ*m^2^ have been shown to elicit neural activation at levels below those determined by the Shannon equation (*k*= 1.85) and the 30 *μ*C cm^−2^ boundary but were found to be damaging to neural tissue [22]. As a result, a new boundary was described for these microelectrodes with a threshold of 4 nC of charge per phase. Furthermore, Shannon himself stated in the seminal 1992 publication that ‘a more comprehensive model of safe levels for electrical stimulation would also account for the effects of pulse rate, pulse duration, stimulus duty cycle, and duration of exposure’ [20]. However, the addition of these other variables turns the simple 2-dimensional Shannon equation into a multi-dimensional problem, that requires more sophisticated algorithms to find a solution.

The use of machine learning algorithms has revolutionized diverse research fields from social sciences to biological sciences and medicine [23–28]. Machine learning applied to the damaging multi-dimensional problem that arises from neural stimulation can capture non-linear relationships between stimulation parameters (features) and neural tissue outcomes (damaging or non-damaging) using training instances from a large database. Subsequently the trained algorithm may be used for prediction on new, untested stimulation parameters (testing instances) [29]. The machine learning approach has been widely used in areas of neuroscience and neural engineering research including brain imaging analysis [30–32], neuroinformatics [33–35], and behavioral analysis [36–38]. Herein, we extend the application of machine learning models to predict electrical stimulation-induced neural tissue damage. Our primary objective was to explore the utility of machine learning to create a more effective tool for estimating safe thresholds for electrical neuromodulation parameters. By utilizing published experimental data and machine learning methods to identify stimulation parameters indicative of tissue damage, we hope to guide the design of additional studies for more targeted investigation of the available parameter space and the underlying mechanisms leading to stimulation-induced tissue damage.

## 2. Materials and methods

### 2.1. Literature search and data collection

For initial data collection, we utilized the bibliographic references included in recent review articles addressing safety of neural stimulation: Cogan *et al* [39], Shepherd and McCreery [40], Miller [41], and McCreery *et al* [12]. We then performed a literature search by generating a comprehensive list of phrases and terms within three key search areas: electrical stimulation of neural tissue, tissue damage, and neural interface device and electrode features. We then identified and removed overlapping or redundant terms and built the final search query in PubMed on 15 February 2023:

(((“Electric Stimulation” [MeSH Terms] OR “Nerve Stimulation” [Text Word] OR “Brain Stimulation” [Text Word] OR “Spinal Cord Stimulation” [Text Word] OR “Microstimulation” [Text Word] OR “Neuromodulation” [Text Word] OR “Stimulation Parameters” [Text Word] OR “Pulsing” [Text Word])) AND ((“Nerve Degeneration” [MeSH Terms] OR “Wallerian Degeneration” [MeSH Terms] OR “Tissue Assessment” [Text Word] OR “Tissue Response” [Text Word] OR “Histopathology” [Text Word] OR “Tissue Damage” [Text Word] OR “Damage” [Text Word] OR “Damaging” [Text Word])) AND ((“Neural Prostheses” [MeSH Terms] OR “Neural Prosthesis” [Text Word] OR “Electrodes” [MeSH Terms] OR “Microelectrodes” [MeSH Terms] OR “Implantable Neurostimulators” [MeSH Terms] OR “Neural Interface” [Text Word] OR “Microelectrode Array” [Text Word] OR “Electroceutical” [Text Word])) AND ((English [Filter]))) NOT (Review[Publication Type])

Inclusion criteria for the literature search were defined as: (1) studies containing primary sources (e.g. experimental data not review articles), (2) studies reporting both electrical stimulation parameters and histopathological assessment of the stimulated neural tissue. Exclusion criteria for the literature search were defined as: (1) review articles or articles containing secondary or tertiary reports of experimental data, (2) studies using non-invasive stimulation, (3) *in vitro* studies, (4) retinal stimulation studies, (5) studies combining electrical stimulation with pharmacological agents (other than antibiotics or those used for pain management), (6) studies that do not report any histopathological assessment.

From each of the included studies, we compiled 12 categorical and 19 numerical features, in addition to an indexing feature (Reference ID). The list of all categorical and numerical features, definitions and units can be found in table 1; a visual explanation of stimulation parameters and waveforms is shown in supplementary figure 1. We included as separate entries all stimulation parameter combinations for articles reporting multiple parameters. For those investigating the same parameters in multiple subjects, we included the average outcome for all subjects as a single entry. All entries were assigned a score to report the histological or functional outcome of the study on a scale from 0 to 4 corresponding to 0—no damage, 1—minimal damage (similar to implanted but unpulsed electrodes), 2—mild damage, 3—moderate damage, and 4—severe damage. Scoring was achieved in one of two ways: (1) as reported on the original assessment—often on the same 0–4 scale but normalized from the reported scale whenever necessary—or scored by two investigators (authors RAF and AGH-R) blinded to each other’s assessments to reduce bias. After all entries were assigned a 0–4 score, binary scores were calculated and assigned to each entry based on the averaged graded responses between investigators: 0—non-damaging, if average graded outcome was less than or equal to one, and 1—damaging, if average graded outcome was greater than one.

**Table 1.** List of features aliases (inputs) and labels (outputs) for our current study.

Feature alias	Type	Description	Units
Reference	Alphanumerical	Citation key to identify reference source.	None
Animal_model	Categorical	Animal model used in cited reference.	None
Nervous_system	Categorical	Label describing if stimulation targeted central or peripheral nervous system.	None
Specific_target	Categorical	Anatomical region targeted for stimulation.	None
Geometry	Categorical	Basic geometric shape of the electrode.	None
Location	Categorical	Implant location within specific target.	None
Material	Categorical	Metallic material composition of the electrode.	None
Charge_mechanism	Categorical	Charge transfer mechanism, defined by the stimulation electrode material.	None
Waveform_type	Categorical	Shape of the waveform used for stimulation.	None
First_ph_direction	Categorical	Direction of the leading phase in the stimulation pulse.	None
Polarity	Categorical	Number of ‘poles’ used for spatial arrangement of stimulation electrodes.	None
Control	Categorical	Label describing if stimulation was voltage-controlled or current-controlled.	None
GSA	Numerical	Geometric surface area of the electrode.	*μ*m^2^
Bias	Mixed	‘None’ if no bias strategy was used, or numerical value of DC voltage (vs. reference) maintained between stimulation pulses.	V
Pulse_width	Numerical	Duration of the leading phase of the stimulation pulse.	*μ*s
Frequency	Numerical	Number of stimulation pulses per second.	Hz
Interphase_delay	Numerical	Duration of time between first and second phase of the stimulation pulse. (monophasic stimulation = 0)	*μ*s
Current	Numerical	Absolute value of the current applied in first phase of the stimulation pulse.	*μ*A
Voltage	Numerical	Absolute value of the voltage applied in first phase of the stimulation pulse.	mV
Charge_per_phase	Numerical	Amount of charge within the first phase of the stimulation pulse.	nC ph^−1^
Charge_density_per_phase	Numerical	Distribution of charge over the electrode surface area within the first phase of the stimulation pulse.	*μ*C ph^−1^ cm^−2^
Current_density	Numerical	Distribution of current over the electrode surface area within the first phase of the stimulation pulse.	mA cm^−2^
Duty_cycle	Numerical	Proportion of time pulsing was on per day.	%
Stim_on	Numerical	Time duration that pulse train was on.	S
Stim_off	Numerical	Time duration that pulse train was off.	S
Stim_total_day	Numerical	Total stimulation duration per day.	S
Days_stim	Numerical	Number of days stimulated during the study.	Days
Stim_total_study	Numerical	Total number of hours of stimulation during the study.	H
Daily_pulses	Numerical	Number of pulses applied per day.	Pulses
Total_pulses	Numerical	Number of pulses applied over the entire study.	Pulses
Daily_accumulated_charge	Numerical	Total amount of charge delivered to the tissue per day.	C
Total_accumulated_charge	Numerical	Total amount of charge per leading phase accumulated to the tissue over the entire study.	C
Damage_level	Numerical	Integer score of 0, 1, 2, 3, or 4.	None
Damage_binary	Binary	Translation of integer scores to binary values, where 0–1 = ‘0’ and 2–4 = ‘1’.	None
*k*	Numerical	Coefficient value calculated using the Shannon equation.	None

### 2.2. Predictions using the Shannon equation

Two features, charge per phase (unit: nC ph^−1^) and charge density per phase (unit: *μ*C cm^−2^ ph^−1^), were used to calculate the *k*-values related to the Shannon equation (equation (1)). We tested a range of *k* threshold values ranging from *k* = −4 to *k* = 4 to obtain a prediction of stimulation outcomes on tissue. The calculated *k*-values below a given threshold (e.g. *k* = 1.85) were classified as non-damaging, and those larger than or equal to the given threshold were classified as damaging [20].

### 2.3. Feature selection and dimensionality reduction

Based on the dataset collected here, we proceeded to identify the feature importance to predict damage resulting from stimulation of neural tissue. First, we used ordinal encoding of categorical features. Then, we used a supervised machine learning random forest algorithm [42] to identify feature importance by measuring how much each feature reduces or increases the accuracy across multiple decision trees. We then proceeded to create three separate feature subsets, each with a different set of features. First, the Shannon feature set included only the charge per phase and charge density features, as previously described for the Shannon equation. Second, a semi-complete feature set included features that are readily available in retrospective studies but are sometimes unpredictable in experimental case-control studies. The features excluded were parameters relating to total stimulation delivered during the study: days, hours, number of pulses, and accumulated charge. In addition, animal model and specific tissue region targeted for stimulation were excluded to enable the creation of a web-based application that is capable of generalizing predictions to the nervous system regardless of experimental design for most user cases. After exclusions, the semi-complete set included 25 features (10 categorical and 15 numerical). Third, a partial feature set based on feature importance, which included the four most important features (revealed by the one-hot random forest algorithm), features required to calculate the values of the four most important features, and features that were possible to calculate thereafter; resulting in the selection of the 13 most important features (1 categorical and 12 numerical) after applying the feature exclusions described above.

Then, for additional visualization, we tested three linear and non-linear dimensionality reduction algorithms [43]: principal component analysis (PCA), *t*-distributed stochastic neighbor embedding (*t*-SNE), and uniform manifold approximation and projection (UMAP).

### 2.4. Machine learning model training and testing

A Dell desktop computer (Intel Core i7-10700 CPU @ 2.90 GHz, Windows 10 enterprise 64-bit OS and 32 GB RAM) and a MacBook Air (3.2 GHz 8-Core Apple M1, macOS Ventura 13 and 16 GB RAM) were used to conduct the machine learning modeling. Scikit–Learn, an open-source Python machine learning library, was used for dimensionality reduction and data visualization (dimension of the embedded space: 2 or 3, all other parameters set as default). Additionally, Scikit–Learn was used to conduct all model training and testing procedures. Other Python libraries, including NumPy, Pandas, and Matplotlib, were also included for data analysis and presentation.

We first transformed categorical features into numerical ones using One Hot encoding [44], which converts each category into a unique binary vector of zeros and ones. We used this method because these features do not have a natural ordered relationship and were of relatively low cardinality (e.g. 4 categories for feature Waveform shape: monophasic, biphasic symmetric, biphasic discharge, biphasic asymmetric) [44]. Next, the complete dataset was randomly split, based on uniform distributions, into training and testing subsets with a ratio of 80:20% (training:testing). The testing subset was withheld from training of the algorithms to ensure that assessment was performed exclusively on previously unseen data. Finally, since the means of numerical features varied significantly across variables, standardization was performed on both training and testing datasets by subtracting from mean and dividing by standard deviation.

For the current study, four supervised learning algorithms were investigated [45, 46]: logistical regression, *k*-nearest neighbor, random forest, and multilayer perceptron (MLP). Logistical regression is a linear model that predicts the probability of a binary outcome based on input features. K-nearest neighbor assigns a label to a new instance based on the majority vote of its closest neighbors. Random forest combines multiple decision trees to produce a robust and accurate prediction. MLP is a type of artificial neural network that can learn non-linear and complex relationships between features and outcomes. Hyperparameters used for each algorithm can be found in supplementary table 1.

A modeling pipeline inspired from our previous study was adopted [45]. First, we screened all models with the One-Hot encoded/standardized training dataset using tenfold cross-validation and adopted two filtering conditions: (1) mean accuracy > 0.75, and (2) standard deviation of accuracy < 0.10. Next, we applied all candidate models on the testing dataset and adopted secondary filtering conditions as: (1) accuracy > 0.85, and (2) AUC (Area Under the ROC Curve) > 0.85.

Furthermore, using the random forest algorithm, we proceeded to train and test the best-performing classifier on the damage level (e.g. 0—no damage; 1—minimal damage; 2—mild damage; 3—moderate damage; and 4—severe damage), rather than the binary outcome. We first performed tenfold cross-validation (number of trees from 1–100) and subsequently applied the trained models on the testing dataset. However, it should be emphasized that due to the increase in cardinality for the output labels, the number of instances for each specific label were even lower than those of binary labeling.

### 2.5. Performance metrics

Performance of each of the different models, including the Shannon equation, was evaluated using threshold dependent and independent metrics, which include:

\begin{align*}\text{Accuracy} &amp; = \frac{{\text{TP} + \text{TN}}}{{\text{TP} + \text{TN} + \text{FP} + \text{FN}}}\end{align*}

\begin{align*}\text{PPV} &amp; = \text{Precision} = \frac{\text{TP}}{{\text{TP} + \text{FP}}}\end{align*}

\begin{align*}\text{TPR} &amp; = \text{Sensitivity} = \frac{\text{TP}}{{\text{TP} + \text{FN}}}\end{align*}

\begin{align*}\text{FPR} &amp; = \frac{\text{FP}}{{\text{FP} + \text{TN}}}\end{align*}

\begin{align*}\text{TNR} &amp; = \text{Specificity} = \frac{\text{TN}}{{\text{TN} + \text{FP}}}\end{align*}

\begin{align*}\text{FNR} &amp; = 1 - \text{TPR}\end{align*}

where TP denotes the number of entries correctly predicted as damaging (true positive), FP denotes the number of entries incorrectly predicted as damaging (false positive), TN denotes entries correctly predicted as non-damaging (true negative), and FN denotes entries incorrectly predicted as non-damaging (false negative). Accuracy (equation (2)) is a metric that calculates the success of correctly predicted cases of all entries. Precision or positive predictive value (PPV; equation (3)) measures the proportion of damaging cases that were actually damaging. The true positive rate (TPR; equation (4)), also known as recall or sensitivity, measures the model’s ability to correctly predict damaging cases from actual cases labeled as damaging. The false positive rate (FPR; equation (5)) measures the model’s capacity to incorrectly predict damaging cases given the actual cases labeled as non-damaging. The true negative rate (TNR; equation (6)) or specificity measures the proportion of non-damaging cases that were actually non-damaging. The false negative rate (FNR; equation (7)) measures the model’s capacity to incorrectly predict non-damaging cases given the actual cases labeled as damaging.

### 2.6. Statistical analysis

Descriptive statistics [47] were calculated for all features and reported as mean ± SEM (Graphpad Prism 10, Version 10.0.2, Graphpad Software, LLC, Boston, MA, USA). First, a Shapiro–Wilk test was used to determine if features were normally distributed. Then, the non-parametric Kolmogorov–Smirnov test was used to reveal statistical differences between damaging and non-damaging parameters when data were not normally distributed. An unpaired *t*-test was used when data were normally distributed. To compare the sensitivity and specificity of each one of the models described here, including the Shannon equation, we used the McNemar test and exact binomial test in RStudio (2023.06.2, Posit Software, PBC, Boston, MA, USA) using the DTComPair package [48].

### 2.7. Web hosting of machine learning algorithm

We designed a Java JSP (JavaServer Pages) based web application for implementing use of the best performing algorithm with the least possible input features. The web application was hosted at Amazon AWS (Amazon web services) using the EC2 t3a.micro instance (https://neurostimml.utdallas.edu).

A secondary literature search with the same keywords was conducted on 28 March 2024, after the machine learning algorithms were trained and tested. The goal of this search was for further validation of the algorithm and testing of the online tool. For this smaller dataset, we collected only the parameters required for the partial machine learning approaches (13 features).

## 3. Results

### 3.1. Dataset of electrical neurostimulation parameters and outcomes

The literature search for neurostimulation parameters and neural tissue outcomes yielded a total of 444 publications in PubMed, and an additional 42 relevant articles were found in literature reviews and books [20, 39, 40]. A total of 58 publications (references in supplementary table 2) were included in the database (supplementary data 1). From these publications, we extracted 385 neuromodulation measurements and their respective histological or functional responses. Responses were scored by the original authors for 60.3% (232) of the included entries; the remainder 39.7% (153) were scored by two independent investigators. There was a correlation of *ρ*= 0.88 between the two investigators, which showed differences in only 36 entries. Binary scores were then calculated based on the averaged graded responses between investigators into damaging and non-damaging. This resulted in 50.7% (195) entries being labeled as non-damaging and 49.3% (190) as damaging, which demonstrated a balanced ratio of nearly 1:1, which is crucial for effective implementation of learning algorithms including Logistic Regression, K-nearest neighbor, Random Forest and Multilayer Perceptron [49, 50].

We graphically depicted the distribution all numerical features in supplementary figure 2 and table 2 shows the median and range for the overall collection, and for damaging and non-damaging entries. In addition, it shows the *p*-values of the Kolmogorov–Smirnov test between both groups for all numerical features. This feature space represents the most comprehensive collection of neuromodulation parameters and tissue outcomes reported to date. There were 15 out of 19 (79%) numerical features that presented statistical differences between non-damaging and damaging parameters. Consistent with the Shannon equation, the charge per phase appeared to have a statistical significance (*p* = 0.0001) between these two groups (damaging: 1.4 ± 0.32 *μ*C ph^−1^ vs. non-damaging: 0.27 ± 0.05 *μ*C ph^−1^); however, the charge density per phase did not reach statistical significance (damaging: 247.7 ± 37.9 *μ*C cm^−2^ ph^−1^ vs. non-damaging: 239.2 ± 39.9 *μ*C cm^−2^ ph^−1^; *p* = 0.07). The mean of the damaging parameters had the following characteristics: larger GSA = 0.04 ± 0.007 cm^2^ (vs. non-damaging: 0.03 ± 0.006 cm^2^; *p* = 0.0002), longer pulse width = 258.8 ± 18.1 *μ*s (vs. non-damaging: 195.0 ± 16.4 *μ*s; *p* = 0.005), similar frequency = 267.1 ± 32.5 Hz (vs. non-damaging: 330.9 ± 38.8 Hz; *p* = 0.07), and higher current = 3.2 ± 0.5 mA (vs. non-damaging: 1.3 ± 0.15 mA; *p* = 0.008). It is interesting to note that there is often an overlap in the median of individual parameters for damaging and non-damaging stimulation, highlighting the non-linearity of the classification problem using individual variables. Furthermore, these results suggest that using only the two variables of the Shannon equation (charge per phase and charge density) to predict stimulation-induced neural tissue damage may not sufficient, and other parameters such as frequency likely may play a determinant role.

**Table 2.** Summary statistics of numerical features included in collection sorted by *p*-value of Kolmogorov–Smirnov test between damaging and non-damaging entries. Note that a value of 0.0 usually represents a value smaller than the number of significant digits.

Feature	Overall Values	Units	Non-damaging	Damaging	*p*-value	
	Median (Min–Max)		Median (Min–Max)	Median (Min–Max)		
Total accumulated charge	3.6 (0.0–6830)	C	0.4 (0.0–6831)	8.5 (0.0–4479)	****	<0.0001
Daily accumulated charge	0.4 (0.0–22.7)	C	0.1 (0.0–7.6)	0.7 (0.0–23)	****	<0.0001
Pulse train ON	4 (0.0–50)	h	1 (0.0–50)	7 (0–50)	****	<0.0001
Stimulation duration per day	10.6 (8–24)	h	7 (0.0–24)	9 (0–24)	***	0.0001
Charge per phase	0.1 (0.0–48)	*μ*C ph^−1^	0.0 (0–6)	0.1 (0.0–48)	***	0.0001
GSA	0.004 (0.0–0.50)	cm^2^	0.002 (0.0–0.5)	0.006 (0.0–0.5)	***	0.0002
Number of pulses per day	0.2 (0.0–22.5)	×10^7^ pulses	0.2 (0.0–22.5)	0.2 (0.0–11.5)	***	0.0007
Voltage amplitude	1.0 (0.0–60)	V	1.0 (0–19)	1.0 (0.0–60)	**	0.002
Total number of pulses	0.009 (0.0–24.2)	×10^9^ pulses	0.01 (5–24.2)	0.5 (0.0–21.6)	**	0.003
Pulse width	150 (25–1000)	*μ*s	110 (25–975)	200 (25–1000)	**	0.005
Current density	272.7 (0.0–32000)	mA cm^−2^	363 (2.5–26084)	273 (0.0–32000)	**	0.007
Current amplitude	1.0 (0.0–48)	mA	1.0 (0–15)	1 (0.0–48)	**	0.008
Total stimulation during study	36 (0.0–51840)	h	36 (0.0–51840)	36 (0.0–38880)	**	0.009
Number of days stimulated	7 (1–2160)	days	18 (1–2160)	4 (1–1620)	*	0.04
Duty cycle	100 (0.0–100)	%	100 (0–100)	100 (1–100)	*	0.04
Frequency	130 (1–2750)	Hz	130 (1–2750)	100 (1–2000)	ns	0.07
Charge density per phase	39.2 (0.3–5216)	*μ*C cm^−2^ ph^−1^	34 (0.3–5217)	71 (0.3–3200)	ns	0.07
Pulse train OFF	0.0 (0.0–24)	h	0.0 (0.0–24)	0 (0–8)	ns	0.45
Interphase delay	0.0 (0.0–800)	*μ*s	0.0 (0–800)	0.0 (0.0–400)	ns	>0.99

### 3.2. Performance assessment of the Shannon equation

**Figure 1.**
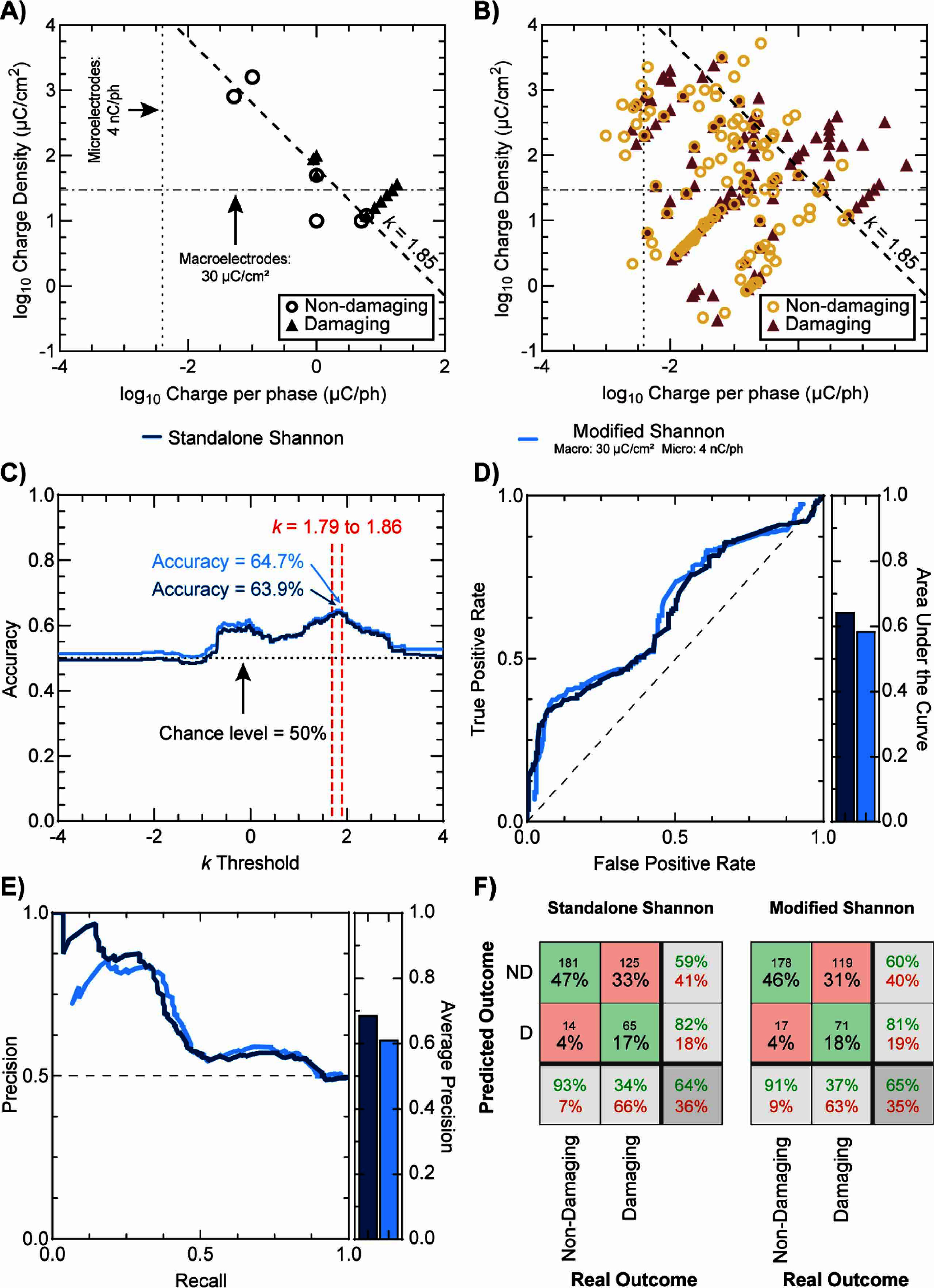
Prediction of electrical stimulation-induced neural tissue outcomes using the Shannon equation. (A) Log-values of charge per phase and charge density per phase showing the dataset used for creation of the Shannon equation. (B) Log-values of charge per phase and charge density per phase showing all data in this collection (including the Shannon equation dataset). (A) & (B) show damaging and non-damaging regions of neuromodulation with a commonly used boundary of *k*= 1.85 for the Shannon equation (dashed line), a charge density per phase for macroelectrodes commonly set at 30 *μ*C cm^−2^ (dash-dotted line), and a maximum charge per phase for microelectrodes commonly set at 4 nC/ph (dotted line). Border only symbols depict non-damaging parameters; filled symbols depict damaging parameters. (C) Accuracies achieved with the standalone Shannon equation (dark blue line) and the modified Shannon equation (30 *μ*C cm^−2^ for macroelectrodes and 4 nC/ph for microelectrodes; light blue) for thresholds between *k* = 0 and *k* = 4. (D) Receiver-operator curves (left) of predictions with the standalone Shannon and modified Shannon equations, and their respective areas under the curve (right). (E) Precision-recall curve (left) for the standalone Shannon and modified Shannon equations, and their respective areas under the curve (right). (F) Confusion matrices for prediction with the standalone Shannon equation (left) and the modified Shannon equation (right) using the best-performing threshold of *k* = 1.85.

Figure 1(A) shows a linear boundary widely accepted to be set at *k* = 1.85 (accepted values range between *k* = 1.5 and *k* = 2.0) that can separate the log-values of charge per phase and charge density for the dataset that gave rise to the Shannon equation. However, plotting the log-values for all entries included in this database (figure 1(B)) revealed that the Shannon equation was not able to linearly separate damaging and non-damaging stimulation parameters. Even when attempting to predict tissue outcomes with the two additional linear boundaries (modified Shannon equation) that are frequently used in combination with the Shannon equation [39]—prediction as non-damaging charge densities up to 30 *μ*C cm^−2^ ph^−1^ for macroelectrodes with GSA > 0.03 cm^2^ (regardless of *k*-value) and a maximum charge per phase of 4 nC ph^−1^ for microelectrodes with GSA < 2000 *μ*m^2^ (regardless of *k*-value)—did not appear to accurately predict electrical stimulation-induced neural tissue outcomes. To fully assess the accuracy of the standalone Shannon equation and the modified Shannon equation, we first calculated the *k*-values for all entries in the collection. Then, we evaluated the accuracies to predict tissue outcomes using different *k*-values ranging from *k* = −4 to *k* = 4.

Consistent with previous observations [39], the maximum accuracy (standalone Shannon: 63.9% and modified Shannon: 64.7%) was achieved with *k*-values ranging from *k* = 1.79 to *k* = 1.86 (figure 1(C)); however, the use of the two boundaries in combination with the Shannon equation did not improve the accuracy. These values were found to be only 14% above the chance level (50%) indicating poor performance of both classifiers and suggesting that both classifiers may not fully capture the complex mechanisms of electrical stimulation-induced neural tissue damage. The performance of both, the standalone Shannon and modified Shannon equations was further investigated, as shown in figures 1(C)–(F), by looking at the receiver-operator (ROC) and precision-recall curves and confusion matrices.

Results show that the Shannon equation has low false positive rates (damaging outcome incorrectly predicted as non-damaging) but also low true positive rates (damaging outcome correctly predicted as damaging) and the area under the curve (AUC) was found to be only slightly above the chance level of 0.50 (standalone Shannon: 0.64 and modified Shannon: 0.59), indicating a poor ability to distinguish between damaging and non-damaging parameters. Furthermore, the precision-recall curves confirm the previous observations (standalone Shannon: 0.68 and modified Shannon: 0.59). Finally, the confusion matrices revealed that both Shannon classifiers using *k* = 1.85 predicted between 63% and 66% of parameters that were damaging as non-damaging, and between 7% and 9% of parameters that were non-damaging as damaging. This suggests that the Shannon equation has a tendency to underestimate potentially damaging stimulating parameters. This could be explained due to the limited feature set used by the Shannon equation (i.e. charge and charge density) that does not take into consideration other variables that may play a role as significant predictors (e.g. pulse width).

### 3.3. Feature selection using ordinal encoding and data visualization to improve prediction of electrical stimulation-induced tissue outcomes

**Figure 2.**
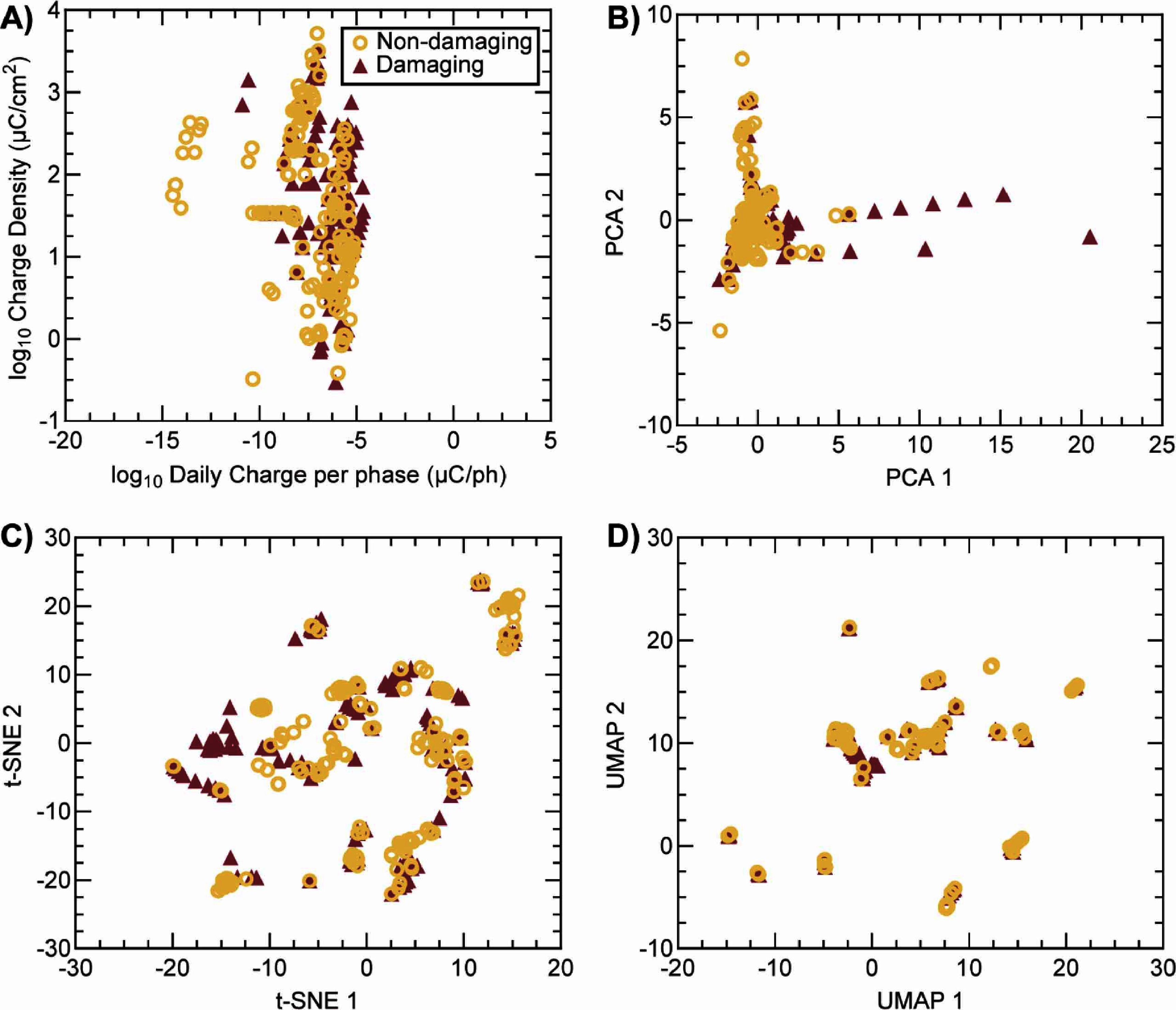
Feature selection and data visualization. Random forest was used to identify features which may be most critical for predicting damage in neural tissues. A threshold of 0.029 for importance score was used for feature selection. (A) Log plot of two of the most important features (charge density vs. daily charge per phase). (B) Visualization of the standardized dataset using PCA. (C) Visualization of the standardized dataset using *t*-SNE. (D) Visualization of the standardized dataset using UMAP. For all plots, border only symbols depict non-damaging parameters; filled symbols depict damaging parameters.

To identify which original features may be most critical for predicting damage in neural tissue, we used a random forest algorithm, where ordinal encoding was used to transform categorical features into numerical formats. Table 3 shows all features sorted by order of importance score. Unsurprisingly, the five most important features were the waveform shape, daily accumulated charge, charge density per phase, charge per phase, and the total number of pulses delivered throughout the study. The five least important features were circuit control (whether stimulation was current- or voltage-controlled), DC bias voltage, interphase delay, charge transfer mechanism, and nervous system (central or peripheral) targeted for stimulation. Then we attempted to find a combination of two features that could more accurately distinguish between damaging and non-damaging stimulation parameters. Figure 2(A) shows the two most important numerical features in a log scale against each other (charge density vs. daily accumulated charge per phase). Supplementary figure 3 shows four additional combinations of the most important features. Results for all feature combinations revealed that there was no possible two-feature linear classifier that could accurately separate damaging and non-damaging parameters. Subsequently, we visualized the data in the collection after attempting three linear and non-linear dimensionality reduction methods (figures 2(B)–(D)): PCA, *t*-distributed stochastic neighbor embedding (*t*-SNE), and uniform manifold approximation and projection (UMAP). However, plots revealed overlapping features between damaging and non-damaging stimulating parameters when visualizing in a 2-dimensional space. Even when visualizing a 3-dimensional space for the principal components (supplementary figure 4), we did not observe a linearly separable feature space. Indeed, the first three principal components from PCA captured relatively low percentages of total variance (26.5%, 15.4% and 12.9%, respectively), implying the necessity of higher dimensional feature spaces. Because of this, we proceeded to train three machine learning approaches capable of non-linear classification: K-nearest Neighbor, Random Forest, and Multi-layer Perceptron.

**Table 3.** Feature importance score for accurate prediction of electrical stimulation-induced tissue outcomes.

Importance	Feature alias	Score
1	Waveform_type	0.07
2	Daily_accumulated_charge	0.05
3	Charge_density	0.05
4	Charge_per_phase	0.05
5	Total_pulses	0.05
6	Specific_target	0.04
7	Total_accumulated_charge	0.04
8	Current_density	0.04
9	Daily_pulses	0.04
10	Stim_total_day	0.04
11	Voltage	0.04
12	Pulse_width	0.04
13	Days_stim	0.04
14	Animal_model	0.04
15	Current	0.04
16	Stim_total_study	0.03
17	Frequency	0.03
18	GSA	0.03
19	Stim_on	0.03
20	Location	0.03
21	Duty_cycle	0.03
22	Material	0.02
23	First_ph_direction	0.02
24	Stim_off	0.02
25	Geometry	0.02
26	Polarity	0.02
27	Nervous_system	0.02
28	Charge_mechanism	0.01
29	Interphase_delay	0.01
30	Bias	0.01
31	Control	0.00

### 3.4. Machine learning approaches using one hot encoding and the Shannon feature set

Standardization of variables in preparation for machine learning training and testing was performed on all numerical features. Then, the training (total: 308; non-damaging: 151 and damaging: 157) and testing (total: 77, non-damaging: 44, and damaging: 33) subsets were created, yielding well-balanced sets between damaging and non-damaging labels (training 1:0.96 and testing 1:1.33, expressed as damaging:non-damaging). Importantly, we emphasize that there was no overlap between training and testing sets. Links to the training and testing datasets, valid machine learning models, performance metrics, and feature subsets can be found in supplementary table 3.

**Figure 3.**
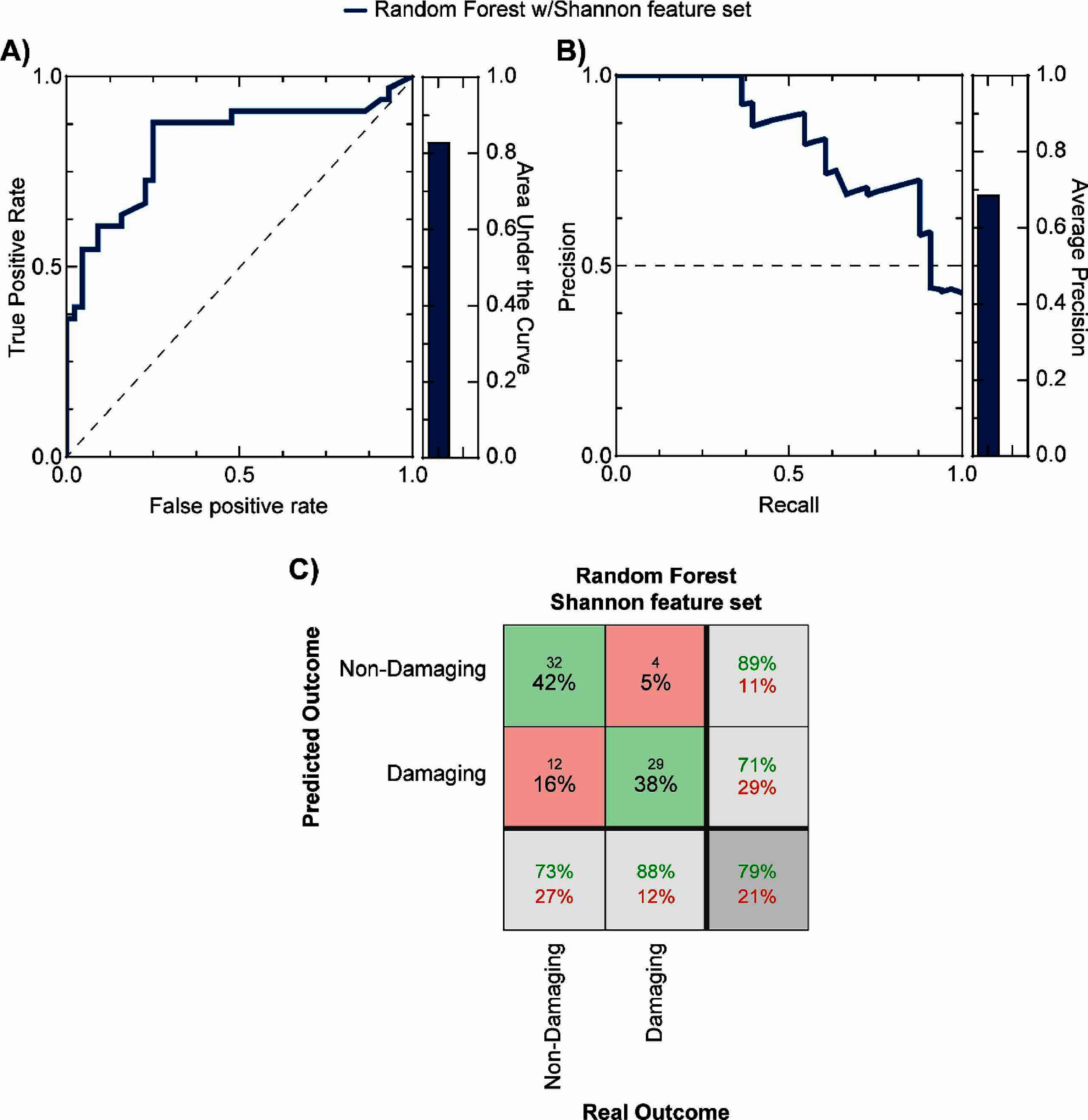
Predictive performance of the candidate machine learning approaches using the Shannon feature set and Random Forest algorithm. (A) shows the receiver-operator curve (left) and the corresponding area under the curve (right) for the Random Forest (dark blue) algorithm. (B) shows the precision-recall curve (left) and corresponding average precision (right) for the algorithm. Confusion matrix for the best-performing classifier that uses the Shannon feature set with random forest algorithm (C) shows high accuracy (79%).

The first machine learning approach used the Shannon feature set with only two features, charge and charge density, to predict stimulation-induced neural tissue outcomes. None of the four algorithms tested (Logistical Regression, K-nearest Neighbor, Random Forest, and Multilayer Perceptron) yielded predictive models which satisfied the first set of filtering conditions during the training stage. However, it is worth noting that when applied to testing data, the best candidate model using the Random Forest algorithm (number of trees: 23, named as RF-Shannon-23) outperformed the modified Shannon equation, as it showed a higher area under the receiver-operator curve (figure 3(A), 0.83 vs 0.59), and similar average precision (figure 3(B), 0.69 vs. 0.61). In addition, compared to the modified Shannon equation (figure 3(C)), this model was found to be more accurate (79% vs. 65%), with a higher ability to correctly predict damaging parameters (TPR = 88% vs. 37%) which resulted in a lower FNR (12% vs. 63%), and a higher TNR (89% vs. 60%). However, this classifier was less precise (71% vs 81%) with higher FPR (27% vs. 9%). The improvement on specificity and sensitivity as denoted by the TNR and TPR, respectively, were both found to be statistically significant (specificity: *p* = 0.005 and sensitivity: *p* = 0.0001). These results in combination with accuracy suggest a moderate improvement on the performance metrics compared to the Shannon equation, and while interesting, we proceeded to investigate the performance metrics of classifiers with the semi-complete and partial feature sets to try to identify a more accurate classifier.

### 3.5. Machine learning approaches using one hot encoding and the semi-complete feature set

**Figure 4.**
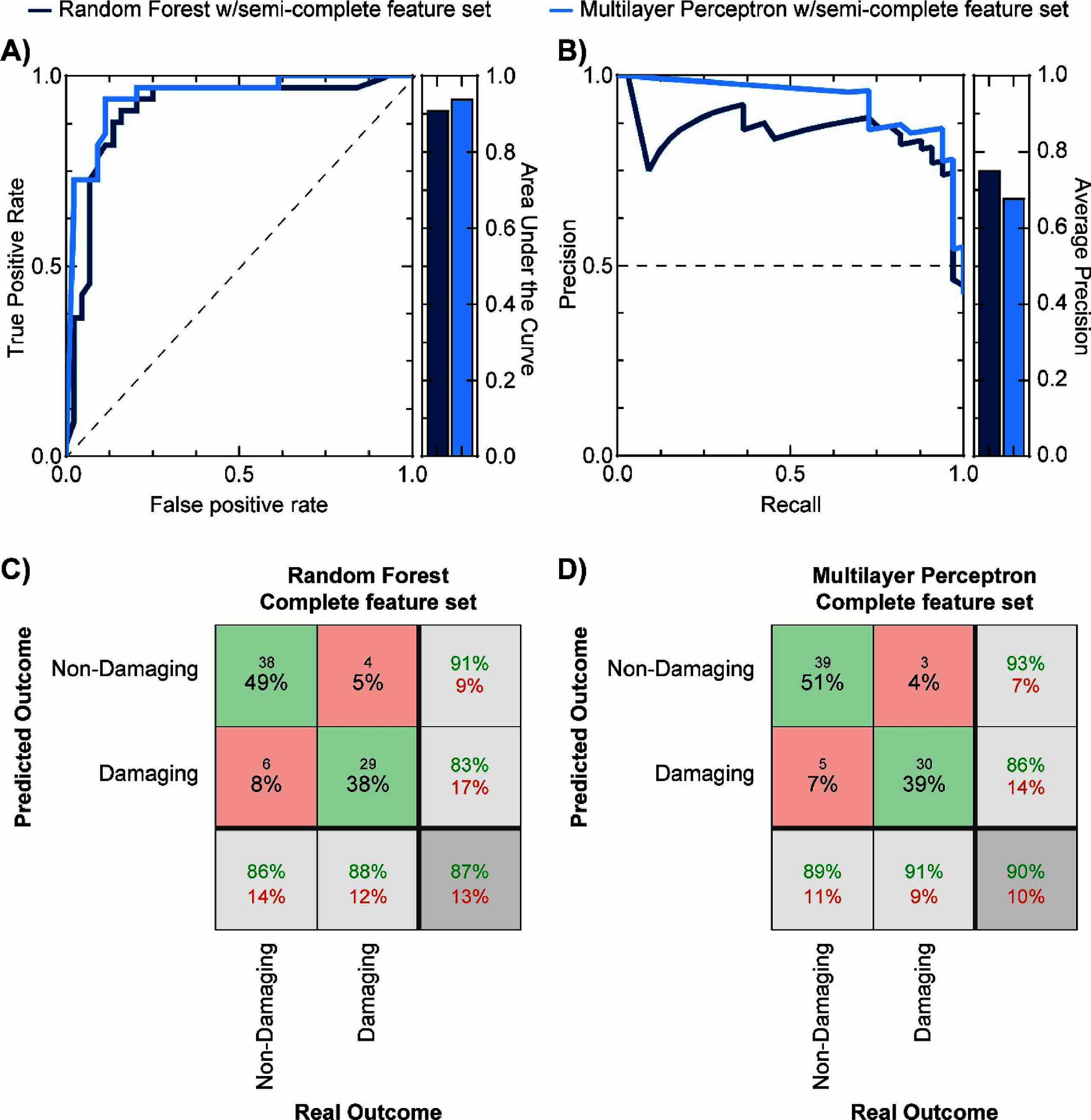
Predictive performance of two machine learning approaches using the semi-complete feature set. (A) shows the receiver-operator curve (left) and the corresponding area under the curve (right) for both the Random Forest (dark blue) and the multilayer perceptron algorithms (light blue). (B) shows the precision-recall curve (left) and corresponding average precision (right) for both algorithms. Confusion matrices for the best-performing classifiers that use the semi-complete feature set with random forest (C) and multilayer perceptron (D) algorithms show high accuracy (87% and 90% respectively) that was higher than the best-performing algorithm using the Shannon feature set (79% with the Random Forest algorithm).

The second machine learning approach used the semi-complete feature set with 25 input features (supplementary table 4) that uses all features except for those that may not be readily available in experimental-control studies (e.g. total stimulation received). Similar to the Shannon feature set, neither Logistical Regression nor K-nearest Neighbor yielded valid predicting models. In contrast, the semi-complete feature set yielded 46 valid models using the Random Forest algorithm, and 445 valid models using the Multilayer Perceptron algorithm. Next, 14 Random Forest models and 46 Multilayer Perceptron models passed secondary filtering. Results for these models are shown in figure 4, where the receiver operator curve with calculation of the area under the curve, and precision-recall curve with the average precision are shown. Results showed that the Random Forest had a lower area under the receiver operator curve (0.91 vs. 0.94) but higher precision (0.75 vs. 0.68) compared to the Multilayer Perceptron. The best-performing models for the Random Forest (number of trees: 89, named as RF-Total-89, accuracy = 87%, precision = 83%, TPR = 88%, FNR = 12%, TNR = 91%, FPR = 14%) and Multilayer Perceptron (number of nodes in first layer: 5, number of nodes in second layer: 26, named as MLP-Total-5–26, accuracy = 90%, precision = 86%, TPR = 91%, FNR = 9%, TNR = 93%, FPR = 11%) had better performance than both the modified Shannon equation and the Random Forest with the Shannon feature set. The specificity of both machine learning models was found to be not-statistically significant (Random Forest: *p* = 0.16 and Multilayer Perceptron: *p* = 0.32), whilst the sensitivity was found to be significantly improved for both models (Random Forest: *p* = 0.0001 and Multilayer Perceptron: *p* < 0.0001). These results support the use of the semi-complete feature set to more accurately predict damaging and non-damaging stimulation parameters. However, some features in this semi-complete set may be hard to estimate for certain stimulation protocols. Because of this, we aimed to further reduce the number of input features needed to predict stimulation-induced outcomes on neural tissue.

### 3.6. Machine learning approaches using one hot encoding and the partial feature set

**Figure 5.**
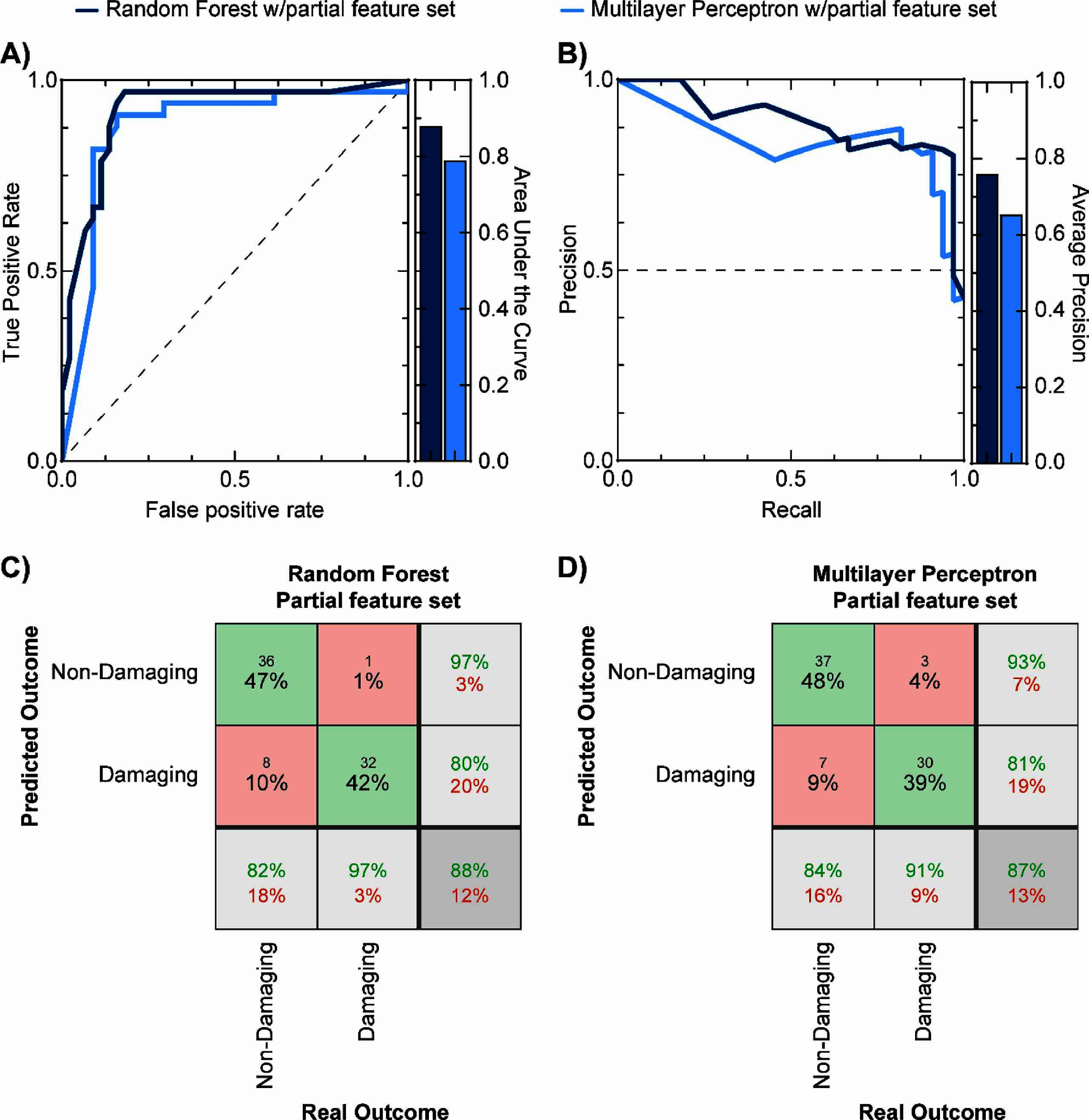
Predictive performance of two candidate machine learning approaches using the partial feature set. (A) shows the receiver-operator curve (left) and the corresponding area under the curve (right) for both the random forest (dark blue) and the multilayer perceptron algorithms (light blue). (B) shows the precision-recall curve (left) and corresponding average precision (right) for both algorithms. Confusion matrices for the best-performing classifiers that use the partial feature set with random forest (C) and multilayer perceptron (D) algorithms show high accuracy (88% and 87%, respectively) that was comparable to the best-performing algorithm with the semi-complete feature set (multilayer perceptron: 90%).

The third machine learning approach used a partial feature set with 13 input features (supplementary table 5) with highest importance scores identified in our feature selection analysis (table 3). Logistic Regression and K-nearest neighbor did not yield any valid predicting models, so they were excluded from further analysis. We found 67 valid models for the partial feature set using the Random Forest algorithm and 720 valid models with the Multilayer Perceptron after the first round of filtering. After secondary filtering, we found 46 valid Random Forest models, and 9 valid Multilayer Perceptron models. Figure 5 shows the performance of these two algorithms, where the Random Forest outperformed the Multilayer perceptron as evidenced by the area under the receiver operator curve (0.92 vs. 0.88, respectively) and the average precision (0.76 vs 0.65). The best-performing models for the Random Forest (number of trees: 19, named as RF-Partial-19, accuracy = 88%, precision = 80%, TPR = 97%, FNR = 3%, TNR = 97%, FPR = 18%) and Multilayer Perceptron (number of nodes in first layer: 10, number of nodes in second layer: 16, named as MLP-Partial-10–16, accuracy = 87%, precision = 81%, TPR = 91%, FNR = 9%, TNR = 93%, FPR = 16%) performed better than the modified Shannon equation and comparably to their counterparts using the semi-complete feature set. Compared to the modified Shannon equation, the specificity of the Random Forest model using the partial feature set was found to be statistically higher (*p* = 0.04) but the Multilayer Perceptron model with this feature set was not significant (*p* = 0.08). The sensitivity for both models was significantly higher than the modified Shannon equation (*p* < 0.0001). We then compared the models with the highest accuracies for the partial feature (Random Forest) set and the semi-complete feature set (Multilayer Perceptron) and found that both specificity (*p* = 0.08) and sensitivity (*p* = 0.16) were not statistically different. These results suggest that the best approach that maximizes accuracy, while minimizing user burden is the use of the partial feature set.

### 3.7. Machine learning approaches using one hot encoding and the partial feature set to predict non-binary damage level

**Figure 6.**
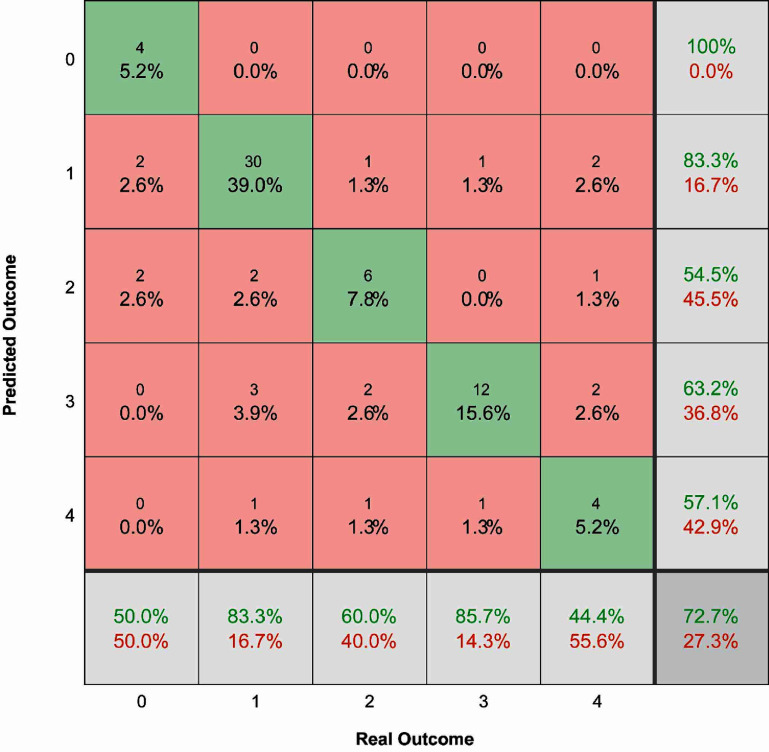
Multiclass confusion matrix for the best-performing Random Forest algorithm with partial feature set for the non-binary damage level, where 0—no damage; 1—minimal damage, 2—mild damage, 3—moderate damage, and 4—severe damage.

Given that the Random Forest algorithm with the partial feature set had the best performance while minimizing the input features required, we proceeded to train a new algorithm to predict damage level (e.g. 0—no damage; 1—minimal damage, 2—mild damage, 3—moderate damage, and 4—severe damage), rather than the binary outcome (1—damaging, 0—non-damaging). The training dataset had an unbalanced ratio with 33 instances labeled as level 0, 124 as level 1, 52 as level 2, 62 as level 3, and 37 as level 4. Similarly, the testing dataset had 8 labeled as level 0, 36 as level 1, 10 as level 2, 14 as level 3, and 9 as level 4. Because of the much smaller sample size, as expected, the overall accuracies were lower than those of the binary analysis. The maximum accuracy (number of trees: 8) achieved for this approach was 73% with an area under the receiver operator curve equal to 0.90 (figure 6). While the performance is better than the Shannon equation, the performance of the binary classifiers was superior and thus, we decided to proceed with the binary classifiers.

### 3.8. Web portal for Random Forest algorithm with partial feature set

**Figure 7.**
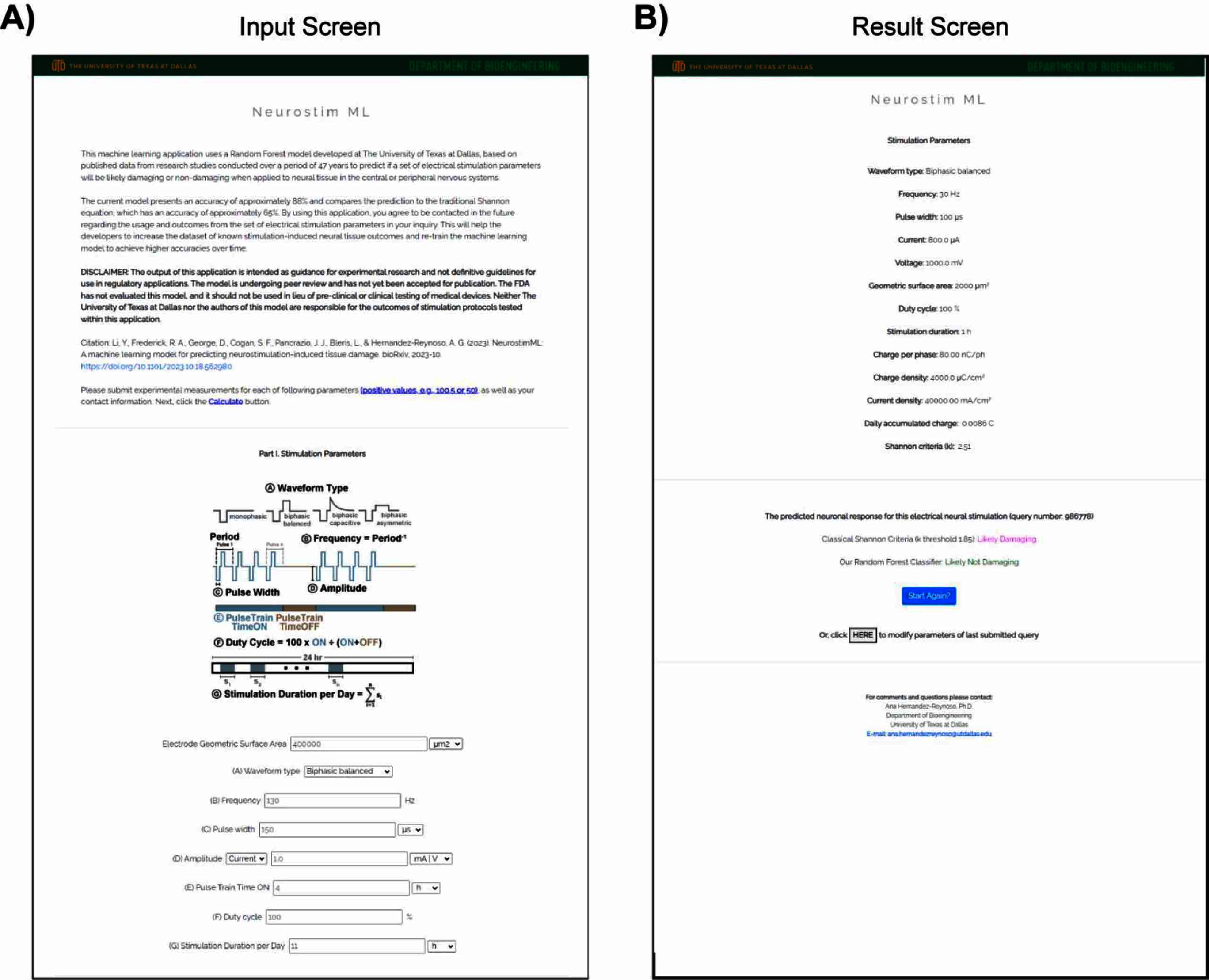
Web portal for Random Forest model using the partial feature set. (A) A sample input page containing the parameter list consisting of waveform shape, frequency, pulse width, current or voltage amplitude, electrode GSA, duty cycle, and stimulation duration. (B) A sample output page showing the predictive results from both our RF-Partial-19 and original Shannon equation (no damage: green, with damage: magenta).

We selected the Random Forest algorithm with the partial feature set (RF-Partial-19) to become publicly available on the web portal (https://neurostimml.utdallas.edu). A screenshot taken from the first version of the web portal can be found in figure 7. The first screen is the submission of experimental condition values for the following parameters (figure 7(A), waveform shape, frequency, pulse width, current or voltage amplitude, electrode GSA, duty cycle, and stimulation duration) from the user, the values for all other features contained within the partial feature set are calculated from the input values and a prediction is made. For output, the predictive results from both our partial and the original Shannon equation are displayed (a sample output is shown in figure 7(B), likely no damage: green, likely tissue damage: magenta).

Validation of the web portal and the trained random forest algorithm with partial feature set was achieved through a secondary literature search, collecting only parameters required as inputs of this trained algorithm. This search resulted in 16 hits ranging from 2023 to 2024. Abstracts were screened, resulting in 4 relevant references being added. In addition, we were able to include two studies from the first search that were originally excluded due to missing input information required for the partial feature set. A total of 27 stimulation parameters (non-damaging: 22 and damaging: 5) were extracted from these 6 publications (supplementary data 9). These values were input into the web portal, and results showed an accuracy of 85%. This value is comparable to the 87% obtained for the testing subset, further validating the trained model.

## 4. Discussion

In the current study, we developed the most comprehensive dataset to-date for stimulation-induced neural tissue outcomes. Then, we developed a random forest-based machine learning classifier with a partial feature set which performed significantly better than the standalone and modified Shannon equations (accuracies: 88.3% and 64%, and 65% respectively). We have made this tool available online for use by the neural engineering field, as well as the complete literature dataset, to aid the generation of safer neural stimulation protocols.

The Shannon equation (standalone and modified) has been used and generally accepted since its inception more than 30 years ago. Here, we have demonstrated that it has a low ability to accurately predict tissue damage outcomes, especially when new electrode designs (e.g. microelectrodes), stimulation parameters (e.g. high frequency stimulation), and tissue regions are targeted for neuromodulation. The Shannon equation achieved a maximum accuracy of 64% to 65%, which is only moderately above the chance level of 50% accuracy. Most of the Shannon equation errors came from stimulation parameters that were predicted as non-damaging but were actually damaging (FNR between 63% to 66%). This is particularly concerning because such mislabeling may result in the clinical use of unsafe stimulation parameters. The use of machine learning approaches, even with the feature set employing only the Shannon input features, resulted in a significant improvement in prediction (FNR ranging between 3% to 12%); however, the partial feature sets yielded the best results (Random Forest: 3% and Multilayer Perceptron: 9%). These results suggest that the model made available for the public (Random Forest with Partial feature set, RF-Partial-19) and used for the construction of the web portal, does not tend to underestimate potentially damaging parameters, and does not significantly overestimate non-damaging parameters (FPR: 18%). Our findings suggest that our predictive model may offer a safer approach for stimulation parameter selection compared to that provided by the Shannon standalone and modified equations.

Selection of input features for the partial set was based on feature importance at a threshold set to reduce the required number of input features, while maintaining a high accuracy. The four features selected as most predictive of stimulation-induced damage are consistent with what has been shown in the literature: waveform shape, daily accumulated charge, charge density and charge per phase. For example, it has been demonstrated that the use of unbalanced, monophasic stimulation pulses may lead to damage of neural tissue during stimulation [51–53]. The remaining three most important features are directly related to the amount of charge either per unit area (e.g. charge density) or per unit of time (e.g. charge per phase and daily accumulated charge), which has been known and accepted for over 30 years [12, 13, 21, 22, 54–56]. Furthermore, the input features of current and voltage amplitude, electrode GSA, pulse width, frequency, duty cycle, and pulse train time, set a baseline value of stimulation ‘intensity’ from which charge density, current density, number of pulses, and daily accumulated charge features were calculated. We believe that including these interconnected features may have improved the accuracy of trained algorithms. This is highlighted when comparing the results of the machine learning Random Forest algorithm using only the Shannon equation features (i.e. charge density and charge per phase), which yielded a maximum accuracy of 79%; and the same Randon Forest algorithm using the Shannon features plus the 11 additional features for the partial set, which improved the accuracy to 88%. It is important to mention that we excluded 6 features ranked as important from the input space (total number of pulses, tissue target structure, total accumulated charge, total days stimulated, animal, and stimulation for total study), which were above the threshold selected for the partial set. This was done because estimating certain features beforehand, like the total number of pulses delivered throughout the entire duration of the study, total accumulated charge, and total number of days stimulated, could be difficult or impossible. Additionally, including the animal model and specific tissue targets in the training set might restrict or make it impossible to use for exploring new targets or for potential human applications. The accuracy of a trained model would probably increase if excluded features were included but would simultaneously limit the usability of the web portal for general users.

Finally, parameters that were less predictive and were not included in the partial feature set are an extension of included parameters for spread of charge delivery over time (interphase delay), identify a spatial arrangement of the electrode array (electrode shape, array configuration), or identify material properties or properties of the method of charge delivery (electrode material, charge transfer mechanism, voltage DC bias). While these parameters were not identified as having high importance in predicting stimulation-induced damage for this particular dataset and model, the inclusion of additional experimental data and exploration of other machine learning based models may later identify some of these parameters as good predictors of stimulation-induced tissue damage. It is important to note that PCA may be done for feature reduction and classifier performance improvement; however, for the dataset presented here, the three principal components captured only 26.5%, 15.4%, and 12.9% of the total variance, indicating that classification using the principal components as input features of a classifier was unlikely to improve the overall classifier performance. Because of this, we focused on feature selection based on ranked importance of features.

It should be noted that due to the nature of neuromodulation studies, our dataset displays two distinct properties: (1) it consisted of both categorical and numerical features; and (2) the dataset was relatively small with only 385 entries. The small dataset size was largely influenced by the fact that acquiring new relevant experimental results is both time-consuming and expensive. Consequently, for each of the proposed analyses, careful consideration was taken for the selection of testing and training subsets. Resulting subsets displayed the same probability distribution compared to the entire dataset by using random training-testing splitting and standardization (using means and standard deviations of numerical features of the training dataset), which is crucial to achieve generalization of the constructed model. Furthermore, it was interesting that for our dataset Random Forest performed slightly better than Multilayer Perceptron algorithms when using the Partial feature set (accuracies for best candidates were 88% and 87%, respectively). Two possible factors could be involved: (1) the nature of Random Forest, which is based on conditional decisions, allows better handling of a mixture of categorical and numerical features [57]; and (2) the relatively small dataset size of our study (total dataset: 385, and training dataset: 308) may be a more pronounced constraint for Multilayer Perceptron, which typically requires large datasets. As an example, using the Partial feature set (16 features), the number of parameters for a relatively simple two-layer MLP (MLP-Partial-10–16) will be 373 (for weights: 16*10 + 10*16 + 16*1 = 336, and for biases: 10 + 16 + 1 = 27), which is greater than the size of our training instances and potentially results in underfitting of the predictive models. Future studies will address the dataset size by expanding our literature search and by using data mining, web crawling algorithms, and other novel methods to automate feature extraction from published studies. However, an immediate challenge with dataset expansion is that published studies are not consistent in reporting stimulating parameters. To standardize reporting of stimulation parameters in neural engineering, we suggest that at least the following parameters are clearly reported in all publications: waveform type, frequency, pulse width, if stimulation is current- or voltage-controlled, pulse amplitude, GSA of the electrodes, duty cycle (or train duration and time between stimulation trains), stimulation duration per day, number of days receiving stimulation, electrode shape, and electrode material.

Next, we noted that for evaluating electrical stimulation-induced tissue damage, a scaled output (e.g. 0—no damage; 1—minimal damage, 2—mild damage, 3—moderate damage, and 4—severe damage) may provide more information compared to the binary labels used to generate models in this study. However, due to the size, and importantly the distribution pattern of the dataset presented here, the accuracy was lower compared to that of the binary classifiers. It was worth noting that most of the errors encountered on the testing subset were only 1 level apart from the true outcome, indicating that if the dataset size is expanded, this approach has the potential to not only distinguish between damaging and non-damaging stimulation parameters, but also to indicate the level of expected damage.

A further limitation of this study was the lack of standardized methods for defining and reporting damaging and non-damaging stimulation parameters in neural tissue across literature. Here, we attempted to mitigate this limitation by having two blinded investigators assign levels based on reported descriptions. Our results suggested an agreement between both investigators, as highlighted by the high correlation between scores (0.88) that resulted in a difference in the binary outcome in less than 10% of entries. Future work can address this limitation by having more than two investigators score the outcomes, and by providing a standard definition of ‘damage’ for the neural engineering field to use in future reports. In addition, the field can benefit from consensus standards by reporting stimulation-induced neural tissue outcomes as reported here from 0 to 4 (0—no damage; 1—minimal damage, 2—mild damage, 3—moderate damage, and 4—severe damage), which are consistent with previously reported outcomes by McCreery [11, 12] and Shepherd [18].

We emphasize that incorporation of additional experimental data would allow further exploration of other modeling methods not tested here (e.g. support vector machines, deep learning) that require higher samples sizes than what was available from the literature search results generated in this study. Including data from parameter values not included in the current database may also lead to the identification of additional features that provide more insight into the relationship between specific stimulation protocols and specific types of tissue damage. In future work, we intend to utilize results from this machine learning based assessment to develop methods for targeted parameter searching and experimental design.

Recent investigations have further pointed out the limitations of the Shannon equation. For example, Vatsyayan and Dayeh [58] expanded the Shannon model in 2022 to account for the electrode site material, electrode size, and inter-electrode spacing. However, their model focuses primarily on electrochemical reactions and still does not incorporate other parameters that have been shown to play a role in the design of safe stimulation protocols. In particular, neither the Shannon nor Vatsyayan–Dayeh models incorporate stimulation parameters related to the cumulative nature or time scale of charge delivery (e.g. frequency or duty cycle). Each model does, however, point to the complexity of the relationship between stimulation parameters and tissue damage. Even within the two-parameter space Shannon model, the features indicating tissue damage exhibit non-linearity as the identification of a boundary between damaging and non-damaging stimulation requires comparing the log_10_ values of both charge per phase and charge density per phase. The Vatsyayan–Dayeh model describes the complex interactions between the non-linear features of electrochemical impedance and charge injection capacity, which are related to material-dependent charge injection mechanisms that can change with stimulation frequency and amplitude.

## 5. Conclusion

Despite the Shannon equation being used and generally accepted for over 30 years, recent investigations have challenged its utility, especially with novel neural interfaces and stimulation waveforms. For this study, we gathered stimulation parameters and neural tissue outcomes from both, systematic and isolated investigations conducted over a period of 47 years, resulting in a dataset of 385 unique stimulation parameter combinations collected from 58 independent studies in a range of animal models and humans, targets in the central and peripheral nervous system, and different electrode materials. This is the most comprehensive dataset available to date and encompasses a wide range of stimulation parameters, including waveform shape, GSA, pulse width, frequency, pulse amplitude, charge per phase, charge density, current density, duty cycle, daily stimulation duration received, daily number of pulses delivered, and daily accumulated charge. This study presents the first machine learning model trained in selecting parameters for neurostimulation experiments and outperforms, by a significant margin, the current gold standard (the Shannon equation). Accordingly, this work has the potential to directly redefine the paradigm in neurostimulation and indirectly revolutionize the neural engineering field. Finally, the user interface may be refined according to end users recommendations, and as additional preclinical and clinical studies with novel stimulation protocols become available, the machine learning models may be retrained to adapt and robustly predict a larger range of stimulation parameters.

## Data Availability

The data that support the findings of this study are openly available at the following URL/DOI: https://github.com/UTDyxl121030/BlerisLab/tree/Neuromodulation/.
